# Comprehensive analysis of expression profile and prognostic significance of interferon regulatory factors in pancreatic cancer

**DOI:** 10.1186/s12863-021-01019-5

**Published:** 2022-01-10

**Authors:** Ke Zhang, Pan-Ling Xu, Yu-Jie Li, Shu Dong, Hui-Feng Gao, Lian-Yu Chen, Hao Chen, Zhen Chen

**Affiliations:** 1 Department of Integrative Oncology, Fudan University Shanghai Cancer Center, Shanghai, 200032 China; 2 Department of Oncology, Shanghai Medical College, Fudan University, Shanghai, 200032 China; 3 Chinese Integrative Medicine Oncology Department, First Affiliated Hospital of Anhui Medical University, Hefei, 230000 Anhui China

**Keywords:** Pancreatic cancer, Bioinformatics analysis, Interference factor, Prognosis, Immune infiltration

## Abstract

**Background:**

Pancreatic cancer (PC) is a highly lethal disease and an increasing cause of cancer-associated mortality worldwide. Interferon regulatory factors (IRFs) play vital roles in immune response and tumor cellular biological processes. However, the specific functions of IRFs in PC and tumor immune response are far from systematically clarified. This study aimed to explorer the expression profile, prognostic significance, and biological function of IRFs in PC.

**Results:**

We observed that the levels of IRF2, 6, 7, 8, and 9 were elevated in tumor compared to normal tissues in PC. IRF7 expression was significantly associated with patients’ pathology stage in PC. PC patients with high IRF2, low IRF3, and high IRF6 levels had significantly poorer overall survival. High mRNA expression, amplification and, deep deletion were the three most common types of genetic alterations of IRFs in PC. Low expression of IRF2, 4, 5, and 8 was resistant to most of the drugs or small molecules from Genomics of Drug Sensitivity in Cancer. Moreover, IRFs were positively correlated with the abundance of tumor infiltrating immune cells in PC, including B cells, CD8+ T cells, CD4+ T cells, macrophages, Neutrophil, and Dendritic cells. Functional analysis indicated that IRFs were involved in T cell receptor signaling pathway, immune response, and Toll-like receptor signaling pathway.

**Conclusions:**

Our results indicated that certain IRFs could serve as potential therapeutic targets and prognostic biomarkers for PC patients. Further basic and clinical studies are needed to validate our findings and generalize the clinical application of IRFs in PC.

**Supplementary Information:**

The online version contains supplementary material available at 10.1186/s12863-021-01019-5.

## Background

Pancreatic cancer (PC) is a lethal disease and ranked as the 14th in cancer incidence and the 7th leading cause of cancer death globally based on the latest data [1]. It is predicted that PC will be the second leading cause of cancer mortality in the USA in the next two or three decades [2]. In total, 60,430 new cases were estimated to be diagnosed with PC, and 48,220 deaths were estimated to happen in the United States in 2021 [3]. PC is hard to detect and diagnose in its early stages due to lacking obvious clinical symptoms and occult location [4]. Approximately, 80-85% patients were diagnosed at advanced stages and not suitable to receive curable surgery. Chemotherapy is currently the standard treatment for these patients. Although target therapy and immunotherapy have achieved promising success in other malignancies, the 5-year survival rate for whole PC patients remains only 10%. These alarming data demonstrated that novel therapeutic targets and prognostic biomarkers are urgent to be discovered.

Interferon regulatory factors (IRFs) family is a variety of transcription factors and it is firstly identified in 1988 [5]. Nine members of the IRF family were presented in mammals (IRF1/2/3/4/5/6/7/8/9). It has been well established that IRFs perform vital functions in innate and adaptive immunity, and immune response [6, 7]. Previous studies also suggested that IRFs played a vital role in the cell biological process of many tumor cells [8]. However, their roles in the regulation of oncogenesis are complex and even controversial based on previous reports. For example, IRF-1 inhibited cell growth in breast cancer by inhibiting NF-κB activity and suppressing TRAF2 and cIAP1 [9]. In gastric cancer, evidence suggested that IRF2 could suppress tumor cell invasion and migration via MMP-1 in STAD [10]. In PC, it is reported that IRF2 expression was upregulated and associated with tumor size, differentiation, pathology stage, and survival of the patients. Knockdown on the expression of IRF2 inhibited cell growth in PC cells [11].

Thus, we embarked on the current study, aiming to explore the expression and its correlation with clinicopathological features of IRFs in PC. Moreover, we also detected the role of IRFs in the immune infiltration in PC and IRFs-associated functions. The results of our study may provide additional data about the function of IRFs in PC and the prognostic and therapeutic biomarkers for PC.

## Results

### Differential expression of IRFs in PC patients

**Fig. 1:**
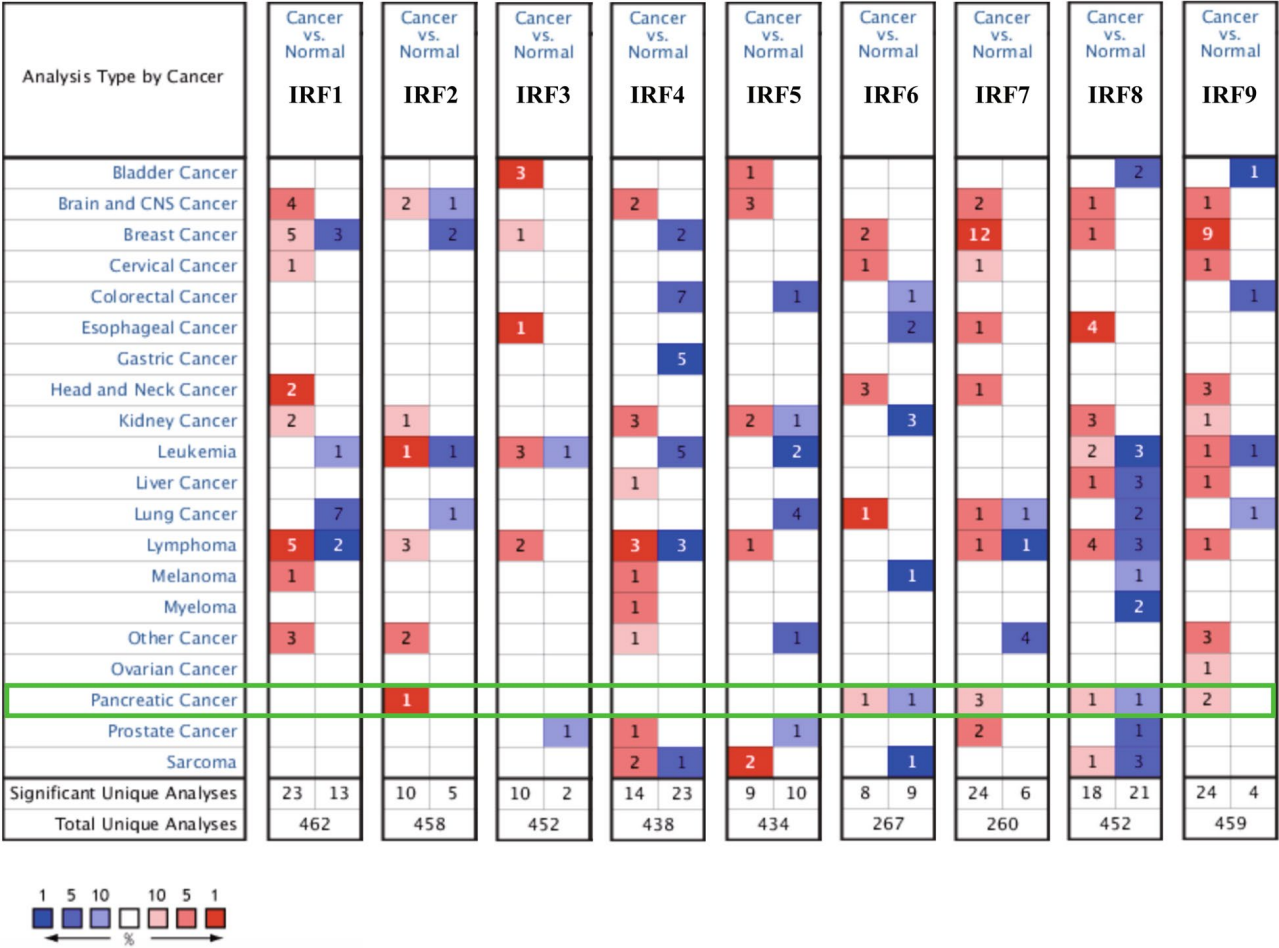
IRFs expression in pancreatic cancer at mRNA level. The number in the figure was the numbers of datasets with statistically significant mRNA over-expression (red) or down-expression (blue) of IRFs, which was obtain with the *P*-value of 0.05 and fold change of 2. This Figure was plotted using ONCOMINE (https://www.oncomine.org/)

**Fig. 2:**
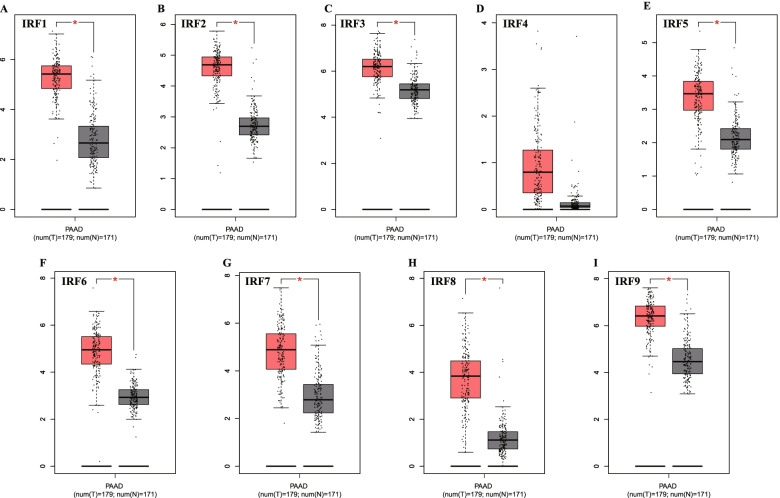
The mRNA level of IRFs in pancreatic cancer. The expression of IRF1 (**A**), IRF2 (**B**), IRF3 (**C**), IRF4 (**D**), IRF5 (**E**), IRF6 (**F**), IRF7 (**G**), IRF8 (**H**), IRF9 (**I**) in pancreatic cancer tissues and normal tissues at mRNA level. This Figure was plotted using GEPIA (http://gepia.cancer-pku.cn/). *P < 0.05; T: tumor tissues; N: normal tissues

We firstly detected the level of IRFs in PC in Oncomine database. The results were shown in Fig. 1 and Table S1. We found that the level of IRF2, IRF6, IRF7, IRF8 and IRF9 were upregulated in tumor tissues in PC (Fig. 1, *P* < 0.05). In addition, we also noticed that no difference was found between tumor tissues and normal tissues about the level of IRF1/3/4/5/6 in PC (Fig. 1)**.** To be more specific, Malte’s dataset revealed that IRF2 expression was increased in Pancreatic Ductal Adenocarcinoma with a fold change (FC) of 2.051 [12]. According to the data of Huadong’s study, IRF6 was upregulated in Pancreatic Carcinoma tissues and the FC is 2.43 [13]. A total of two datasets demonstrated the upregulation of IRF7 in PC [12, 14]. Moreover, three datasets suggested that IRF8 expression was increased in PC [15–17]. We also found that the level of IRF9 was elevated in PC with the FC of 2.205 and 2095 [13, 17]. This is followed by the verification of the expression of IRFs in PC using the TCGA dataset. We found that the mRNA level of IRF1, IRF2, IRF3, IRF5, IRF6, IRF7, IRF8 and IRF9 (Fig. 2A-I**)** were upregulated in PC (All *p* < 0.05). Therefore, we suggested that the level of IRF3, IRF6, IRF7, IRF8 and IRF9 were upregulated in tumor tissues of PC.

**Fig. 3:**
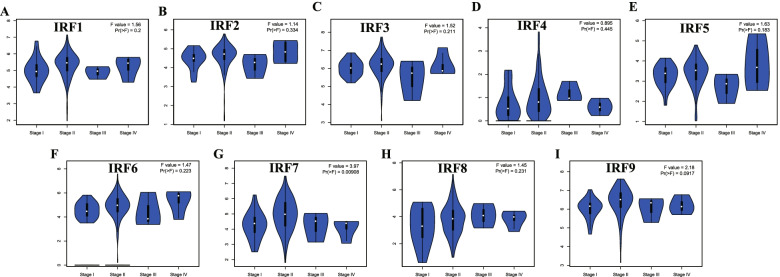
Correlation between IRFs and the pathological stage of pancreatic cancer patients. The expression of IRF1 (**A**), IRF2 (**B**), IRF3 (**C**), IRF4 (**D**), IRF5 (**E**), IRF6 (**F**), IRF7 (**G**), IRF8 (**H**), IRF9 (**I**) in different pathological stage of pancreatic cancer patients at mRNA level. This Figure was plotted using GEPIA (http://gepia.cancer-pku.cn/). *P < 0.05

The association between the level of IRFs and patient’s pathology stage in PC were also detected. Interestingly, a significant association was obtained between IRF7 expression and patient’s pathology stage in PC (Fig. 3G, *p* < 0.00908). Further analysis showed that the expression of IRF7 is significantly higher in stage II compared with stage I (*p* = 0.014). However, there was no association between IRF1/2/3/4/5/6/8/9 expression and patient’s pathology stage in PC (Fig. 3, *p* > 0.05).

### Prognostic value of IRFs in PC patients

**Fig. 4:**
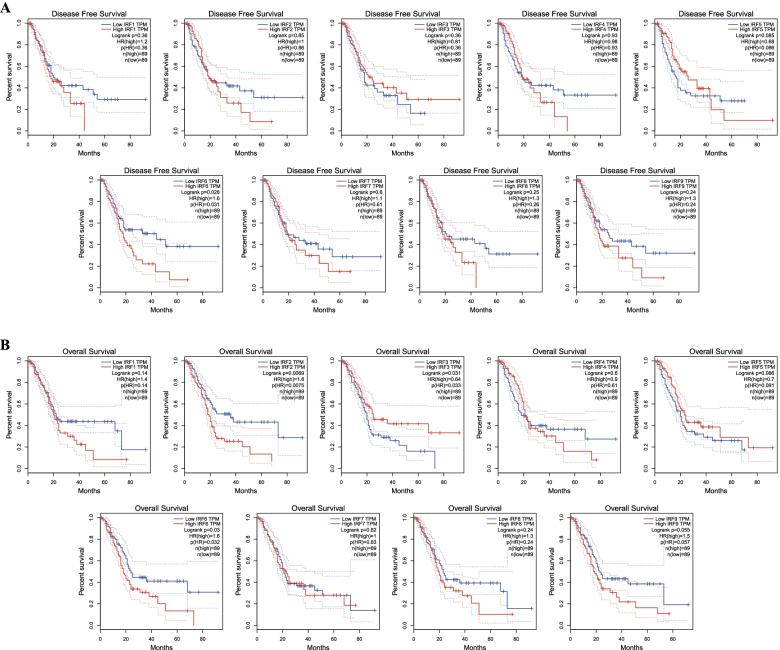
The prognostic value of IRFs in pancreatic cancer. **A** The overall survival of pancreatic cancer patients with high/low mRNA level of IRFs. **B** The disease-free survival of pancreatic cancer patients with high/low mRNA level of IRFs. All the analyses were performed with Kaplan-Meier analysis. This Figure was plotted using GEPIA (http://gepia.cancer-pku.cn/). HR: Hazard Ratio

The prognostic value of IRFs in PC was explored using TCGA dataset. The data showed that PC patients with high IRF2 (HR = 1.8, *p* = 0.0069) and low IRF3 expression (HR = 1.6, *p* = 0.031) were associated with poor overall survival (Fig. 4A). Particularly, PC patients with high IRF6 expression had both poor overall survival (HR = 1.6, *p* = 0.03) (Fig. 4A) and poor disease-free survival (HR = 1.6, *p* = 0.028) (Fig. 4B).

### Co-expression, genetic alteration, and drug sensitivity analyses of IRFs in PC patients

**Fig. 5:**
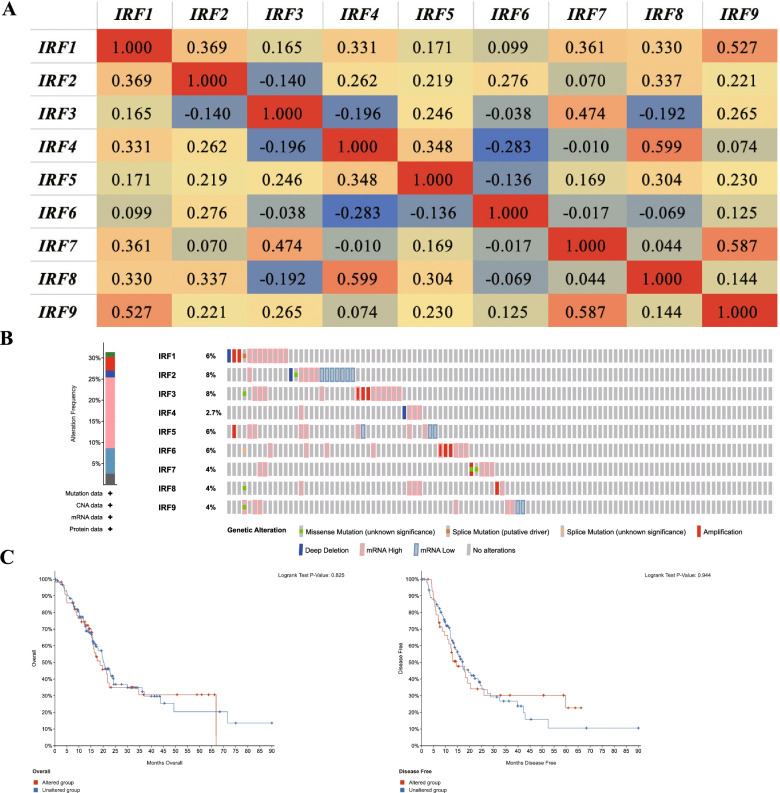
Co-expression and genetic alteration of IRFs in pancreatic cancer. **A** Correlation heat map of each member of IRFs in pancreatic cancer. **B** Summary of genetic alterations of IRFs in pancreatic cancer. **C** Overall survival and disease-free survival of pancreatic cancer patients with/without IRFs genetic alterations. This Figure was plotted using cBioportal (https://www.cbioportal.org/)

Comprehensive analyses were performed to explore the molecular character of IRFs in PC using cBioportal. There was a low to moderate correlation among the mRNA level of each IRFs member in patients with PC (Fig. 5A). Moreover, the genetic alterations analysis revealed that IRF1, IRF2, IRF3, IRF4, IRF5, IRF6, IRF7, IRF8 and IRF9 were altered in 6, 8, 8, 2.7, 6, 6, 4, 4, and 4% of the queried PC samples, respectively (Fig. 5B). High mRNA expression, amplification and deep deletion were the three most common type of genetic alterations in these samples (Fig. 5B). To clarify whether these genetic alterations could affect the prognosis of PC patients. Kaplan-Meier method was drawn and revealed that genetic alterations of IRFs could not affect the overall survival and disease-free survival of PC patients (Fig. 5C, *p* > 0.05). Drug sensitivity analysis was also performed. And the results suggested that low expression of IRF2/4/5/8 were resistant to most of the drugs or small molecules from GDSC (Fig. S1).

### Immune cell infiltration analysis of IRFs in PC patients

**Fig. 6:**
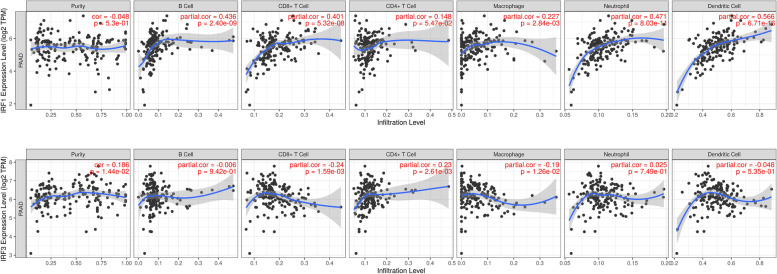
The correlation between IRFs and immune infiltration in pancreatic cancer. The correlation between the expression of IRF1 (**A**), IRF2 (**B**), IRF3 (**C**), IRF4 (**D**), IRF5 (**E**), IRF6 (**F**), IRF7 (**G**), IRF8 (**H**), IRF9 (**I**) and the abundance of B cells, CD8+ T cells, CD4+ T cells, Macrophage, Neutrophils and Dendritic cells. This Figure was plotted using TIMER (https://cistrome.shinyapps.io/timer/)

Tumor-infiltrating lymphocytes could serve as a biomarker for predicting sentinel lymph node status and cancer patients’ survival [18, 19]. The previous study has revealed close correlation between immune infiltration analysis and IRFs in cancers [20]. In our study, a comprehensive detection of the correlation between IRFs and immune cell infiltration in PC was conducted using TIMER. As shown in Fig. 6, the level of IRF7 was positively associated with the infiltration abundance of B cells (Cor = 0.436, *P* = 2.40e-09), CD8+ T cells (Cor = 0.401, *P* = 5.32e-08) macrophages (Cor = 0.227, *P* = 2.84e-3), Neutrophils (Cor = 0.471, *P* = 8.03e-11) and Dendritic cells (Cor = 0.566, *P* = 6.71e-16) (Fig. 6A). Interestingly, the expression of IRF2 and IRF6 also showed a positive association with the infiltration abundance of these five immune cells in PC (Fig. 6B and F**,** all *p* < 0.05**)**. As for IRF3, a positive correlation was obtained between IRF3 expression and the infiltration abundance of B cells, CD8+ T cells and CD4+ T cells (Fig. 6C). Moreover, the expression of IRF4 (Fig. 6D), IRF5 (Fig. 6E), IRF8 (Fig. 6H) and IRF9(Fig. 6I) was positively associated with all these six immune cells, including B cells, CD8+ T cells, CD4+ T cells, macrophages, Neutrophils and Dendritic cells (all *p* < 0.05). We also found that IRF7 expression was associated with the infiltration abundance of CD8+ T cells (Cor = − 0.209, *P* = 6.07e-083), CD4+ T cells (Cor = 0.389, *P* = 1.77e-7), Neutrophils (Cor = 0.252, *P* = 8.72e-4) **(**Fig. 6G). We also explored the effect of copy number alteration of IRF on the immune cell infiltration in PC. As a result, copy number alteration of IRF could suppress the infiltration level of immune cells to some extent (Fig. S2).

### IRFs-associated biologic functions in PC

**Fig. 7:**
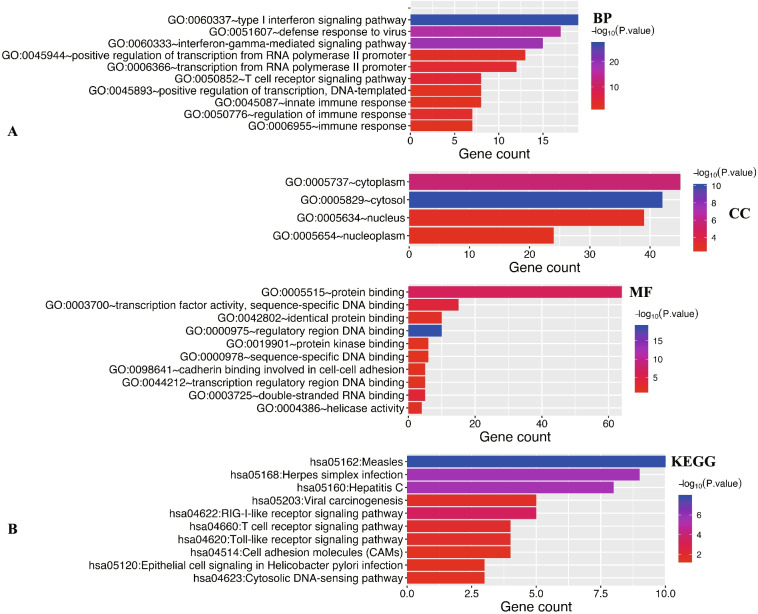
The enrichment analysis of IRFs and neighboring genes. **A** Bar plot of GO enrichment in cellular component terms, biological process terms, and molecular function terms. **B** Bar plot of KEGG enriched terms. This Figure was plotted using David 6.8 (https://david.ncifcrf.gov/home.jsp)

DAVID 6.8 and Metascape were utilized to explore the biological functions of IRFs and their neighboring genes (Table S2) in PC. As we could see in Fig. 7 the results of functional analysis obtained from DAVID 6.8. The item of GO enrichment analysis revealed that IRFs and their neighboring genes were mainly involved in defense response to virus, T cell receptor signaling pathway, immune response, regulatory region DNA binding, protein binding, sequence-specific DNA binding, transcription factor activity, sequence-specific DNA binding, cadherin binding involved in cell-cell adhesion and type I interferon signaling pathway (Fig. 7A). The item of KEGG pathway revealed that IRFs and their neighboring genes were mainly linked to RIG-I-like receptor signaling pathway, T cell receptor signaling pathway, Toll-like receptor signaling pathway, Cell adhesion molecules (CAMs) and Cytosolic DNA-sensing pathway (Fig. 7B). PPI network showed that IRFs were mainly involved in immune response, sequence-specific DNA binding, response to Type I interferon (Fig. S3**)**.

To further detect IRFs-associated functions in patients with PC, Metascape was further used to perform enrichment analysis. Interestingly, the result suggested that IRFs and their neighboring genes were mainly linked to regulation of cytokine production, immune response-activating signal transduction in GO function analysis and type I interferon signaling pathway (Fig. S4A and B, Table S3). The data of KEGG pathways analyses were shown in Fig. S4C, D, and Table S4. As expected, IRFs and their neighboring genes were involved in T cell receptor signaling pathway, Cell adhesion molecules (CAMs), Antigen processing (presentation) and Hippo signaling pathway. Moreover, PPI network and Molecular Complex Detection (MCODE) components were isolated to identify the correlation between IRFs and their neighboring genes. The result indicated the involvement of IRFs in T cell receptor signaling pathway and Pertussis (Fig. S4E and F).

## Discussion

Increasing researches have reported the significant functions of IRFs in immune response [21]. IRFs also exert an important function in basic cellular mechanisms, including cell invasion, proliferation, and apoptosis [22, 23]. Moreover, IRFs were also involved in the tumorigenesis and progression of cancers, including colorectal cancer, hepatocellular carcinoma, and esophageal cancer [24–26]. In this study, we conducted a comprehensive analysis to explore the specific role of IRFs in PC.

We first detected the mRNA level of IRFs in PC, revealing that the level of IRF2, IRF6, IRF7, IRF8 and IRF9 were elevated in tumor tissues in PC. Further prognosis analysis revealed that high IRF2 expression, low IRF3 expression, and high IRF6 predict poor survival in PC. Similarly, IRFs were also suggested to be prognosis biomarkers in various malignancies. It was reported that low IRF3 was associated with poor disease free survival and overall survival in urothelial carcinoma [27]. Another study indicated high IRF2 expression independently predicts poor overall survival in colorectal cancer [28]. These two were consistent with our study. Moreover, IRF3 and IRF7 were linked to a poor prognosis in colon adenocarcinoma [20].

Another significant finding is that IRFs were correlated with the abundance of immune cells in PC, including B cells, CD8+ T cells, CD4+ T cells, macrophages, Neutrophil and Dendritic cells. In fact, these immune cells have been proved to be biomarker or involved in the tumor progression of PC microenvironment. Mobilization of CD8 + T Cells could promote PD-1 checkpoint therapy in human PC by blockading CXCR4 [29]. Another study suggested infiltrating CD4/CD8 high T cells as a biomarker involved in good prognosis in PC [30]. Neutrophil extracellular traps could facilitate liver micro metastasis by activating cancer-associated fibroblasts in PC [31]. Moreover, dendritic cell paucity could result in dysfunctional immune surveillance in PC [32].

Enrichment analysis was performed, which revealed that IRFs and their neighboring genes mainly associated with T cell receptor signaling pathway, immune response, Toll-like receptor signaling pathway, Cell adhesion molecules (CAMs), sequence-specific DNA binding, response to Type I interferon, and Hippo signaling pathway. Interestingly, Toll-like receptor signaling pathway was associated with immune response and play an important function in cancer initiation and progression [33, 34]. CAMs play a vital role in cancer progression and metastasis [35]. Increasing studies revealed that T cell receptor signaling was involved in the control of regulatory T cell differentiation and function, which plays an important function in cancer initiation and progression [36].

Based on our results, we would like to emphasize the potential roles of IRF2, IRF3, and IRF6. Generally, our finding suggested that IRF2 functions as an oncoprotein, which is consistent with previous studies. IRF2 expression was increased in esophageal squamous cell carcinomas (ESCC) compared with matched normal esophageal tissues. In addition, the tumorigenicity of ESCC cells was enhanced with IRF2 overexpression in nude mice model [37]. IRF2 could attenuated apoptosis through induction of autophagy in acute myelocytic leukemia cells [38]. A recent study found that Kras-IRF2 axis drives immune suppression and immune therapy resistance in colorectal cancer [39]. Particularly, our finding was supported by a previous study which reported that IRF2 expression was upregulated and associated with tumor size, differentiation, pathology stage, and survival of PC patients and knockdown on the expression of IRF2 inhibited cell growth in PC cells [11]. Evidence above suggests that IRF2 is a potential biomarker and therapeutic target in PC and other malignancies.

IRF3 was reported to participant in the innate immune response against cancer via STING pathway [40]. A recent study revealed that IRF3 prevents colorectal tumorigenesis via inhibiting the nuclear translocation of β-catenin. Moreover, high expression of IRF3 correlated with favorable survival in colorectal cancer, lung adenocarcinoma, and hepatocellular carcinoma patients [41]. Consistent with the literature above, our results showed that IRF3 expression positively correlated with the infiltration abundance of B cells, CD8+ T cells and CD4+ T cells. Besides, high IRF3 expression level is associated with better survival. These results indicated that IRF3 functions as a tumor suppressor.

Our results showed that IRF6 was overexpressed in PC compared with normal tissue and high expression level of IRF6 corelated with poor survival. It seems that IRF6 plays a pro-cancer role and is a promising therapeutic target in PC. However, previous studies indicated that IRF6 acts as a tumor suppressor [42, 43]. And the decreased expression of IRF6 was clinically correlated with poor prognosis of Gastric cancer [44]. Our findings are contrary to previous studies which have suggested further experimental and clinical research to clarify the roles of IRF6 in PC.

Some limitations must be reported about our study. Firstly, most analyses were performed at mRNA level but not protein level and gene level. Secondly, immune suppressive cells, such as regulatory T cells (Tregs) and myeloid-derived suppressor cells (MDSCs) also defines the microenvironment of PC [45]. These immune suppressive cells may contribute to tumor progression and poor survival. Unfortunately, relevant data are temporarily unavailable. Furthermore, it would be better to validate our results by performing in vivo and in vitro experiments.

## Conclusion

This study comprehensively explored the expression profile, prognostic value, and biological functions of IRF family members in PC, providing insights of IRFs as potential therapeutic targets and prognostic biomarker for PC. Further basic and clinical studies are needed to validate our findings and generalize the clinical application of IRFs in PC.

## Methods

### ONCOMINE

ONCOMINE (https://www.oncomine.org/) is an online platform including oncogene expression signatures from over 80,000 cancer samples [46]. We can analyze the mRNA level of target genes in cancer and normal tissues by using ONCOMINE database and the *p*-value was 0.05, the fold change was 2 and the gene rank was10%, we analyzed the mRNA level of IRFs in PC and normal tissue with student’s t-test.

### GEPIA

GEPIA (http://gepia.cancer-pku.cn/) is a novel web portal collecting mRNA data from The Cancer Genome Atlas (TCGA) database [47]. A total of 186 complete TCGA PC samples were involved in the following analyses. we further detected the mRNA level of IRFs in PC. Setting the group cutoff as median, we explored the prognostic value of IRFs in PC by using overall survival (OS) plots and disease-free survival (DFS) plots. Hazard ratio (HR) and log-rank *P*-value were also listed in the plots. Moreover, correlation analysis was conducted to explore the genes most associated with each member of IRFs in PC.

### cBioPortal

cBioPortal (https://www.cbioportal.org/) is a comprehensive web portal that integrates genomic data from over 30,000 cancer samples of various cancer types [48]. Using the TCGA datasets (*N* = 186), we performed gene alterations analysis of IRFs in PC samples, which was summarized by the “Oncoprint” module. Using cBioportal, we also performed co-expression among IRFs in PC samples in the “Co-expression” module with spearman’s correlation. In addition, we set a threshold as ±2.0 in mRNA expression z-scores (RNA Seq V2 RSEM) and protein expression z-scores (RPPA). Putative copy-number determined using GISTIC 2.0.

### GSCALite

GSCALite (http://bioinfo.life.hust.edu.cn/web/GSCALite/) is a novel web portal collecting mRNA data from the TCGA database [49]. In drug sensitivity analysis, the association between IRFs level and the drug using the data from GDSC (Genomics of Drug Sensitivity in Cancer) was analyzed with the spearman correlation. The positive correlation means that the gene high expression is resistant to the drug, vise verse. These analyses were performed with TCGA datasets (*N* = 186) and a *p*-value < 0.05 indicates statistical significance.

### TIMER

TIMER (https://cistrome.shinyapps.io/timer/) is a web server for comprehensively analysis the relationship between immune cells infiltration and gene expression [50]. In the current study, we first evaluated the association between IRFs expression in PC and abundance of B cell, CD8+ T cell, CD4+ T cell, Macrophage, Neutrophil, and Dendritic cell according to TCGA datasets (*N* = 186). In the “SCNA” module, we performed the comparison of tumor infiltration levels among tumors with different somatic copy number alterations of IRFs. A *P*-value of less than 0.05 meant significant difference existed.

### David 6.8

DAVID 6.8 (https://david.ncifcrf.gov/home.jsp) is a functional annotation tool providing the biological function of submitted genes [51]. After isolated the genes most associated with each member of IRFs in pancreatic adenocarcinoma, we performed ene Ontology (GO) [52, 53] and Kyoto Encyclopedia of Genes and Genomes (KEGG) [54–56] pathway enrichment analysis of these genes and the result was visualized with R project using a “ggplot2” package and a *p* < 0.05.

### GeneMANIA

GeneMANIA (http://genemania.org/) is established to predict the biological functions of target gene sets [57]. Protein protein interaction (PPI) networks of the IRFs were constructed to indicate the relative relationships and the potential functions of these gene sets.

### Metascape

Metascape (http://metascape.org) is a reliable functional annotation tool providing the biological function of submitted genes [58]. Based on the functional annotation of gene/protein lists, Metascape can facilitate data-driven decisions. After isolated the genes most associated with each member of IRFs in pancreatic adenocarcinoma, we further explored the function of IRFs and closely related neighbor genes.

## Supplementary Information

**Additional file 1.**

## Data Availability

All data generated or analyzed during this study are included in the article and its supplementary information files. The dataset supporting the conclusions of this article is available in the TCGA repository, project identifier ‘TCGA-PAAD’ and hyperlink to dataset in https://portal.gdc.cancer.gov/repository.

## Abbreviations

CAMs: Cell adhesion molecules
CD: Cluster of differentiation
DFS: Disease-free survival
GO: Gene ontology
HR: Hazard ratio
IRF: Interferon regulatory factor
KEGG: Kyoto Encyclopedia of Genes and Genomes
MCODE: Molecular Complex Detection
OS: Overall survival
PC: Pancreatic cancer
PD-1: Programmed death-1
PPI: Protein-protein interaction
TCGA: The Cancer Genome Atlas
