# Supplementary material for: Comprehensive analysis of expression profile and prognostic significance of interferon regulatory factors in pancreatic cancer
Source: BMC Genom Data. 2022 Jan 10;23:5. doi: 10.1186/s12863-021-01019-5 (PMC8751298; doi:10.1186/s12863-021-01019-5)
Supplement: Supplementary file 1 — Additional file 1. [file 12863_2021_1019_MOESM1_ESM.docx]

Table S1. The mRNA levels of IRFs in pancreatic carcinoma (Oncomine).

| **IRFs** | **Types of pancreatic carcinoma vs. normal** | **Fold Change** | **P value** | **t-test** | **PMID** |
| --- | --- | --- | --- | --- | --- |
| IRF1  IRF2 | NA  Pancreatic Ductal Adenocarcinoma | NA  2.051 | NA  2.05E-6 | NA  9.336 | NA  16103885 |
| IRF3 | NA | NA | NA | NA | NA |
| IRF4 | NA | NA | NA | NA | NA |
| IRF5 | NA | NA | NA | NA | NA |
| IRF6 | Pancreatic Carcinoma | 2.430 | 1.32E-4 | 4.250 | 19732725 |
| IRF7 | Pancreatic Adenocarcinoma  Pancreatic Ductal Adenocarcinoma | 374.440  2.633 | 0.001  0.002 | 6.168  3.491 | 12750293  16103885 |
| IRF8 | Pancreatic Carcinoma  Pancreatic Adenocarcinoma  Pancreatic Ductal Adenocarcinoma | 1.949  2.838  1.975 | 2.43E-4  0.005  0.039 | 4.524  3.324  1.875 | 15867264  12651607  15548371 |
| IRF9 | Pancreatic Ductal Adenocarcinoma  Pancreatic Carcinoma | 2.205  2.095 | 2.30E-8  1.37E-4 | 6.424  4.348 | 19260470  19732725 |

Table S2. The top 10 significant gene correlated with IRFs in pancreatic carcinoma (GEPIA).

| **IRFs** | | **Correlated genes** |
| --- | --- | --- |
| IRF1  IRF2  IRF3  IRF4  IRF5  IRF6  IRF7  IRF8  IRF9 | GBP4, UBE2L6, B2M, GBP1, PSMB9, PSME1, PARP14, TAP1, TRAFD1, ETV7  MAT2B, CTCF, CASP3, MOB1A, NFKB1, EFCAB14, UVRAG, FBXO38, ELF1, ABI1  PNKP, PRKD2, PIH1D1, NOSIP, ALDH16A1, DHX34, EPS8L1, CTC-479C5.10, SNHG12, MICALL2  IKZF1, SASH3, IKZF3, LY9, TESPA1, P2RY10, ITGAL, SIT1, TRAC, CD247  RGS19, HSPBAP1, FGD2, ADPGK, KCNAB2, GMIP, DOK3, PARVG, C2CD2, TNRC6C-AS1  TP73, F11R, INADL, FAM83H-AS1, C1orf74, CTNNBIP1, ESRP1, CAST, L1RAP, CTNND1  IFI44, DHX58, ISG15, NRIR, IFI6, IFITM1, IFI35, MX1, MOV10, IFITM3  P2RY10, KBTBD8, IKZF1, RCSD1, IKZF3, PTPRC, CD28, TAGAP, ARHGAP25, GVINP1  OAS2, IFIT3, CMPK2, PARP14, PSME1, OAS1, IFI44L, TRIM21, DDX60, OAS3 | |

Table S3. The GO function analysis of IRFs and neighbor genes in pancreatic carcinoma (Metascape).

| GO | Category | Description | Count | % | Log10(P) | Log10(q) |
| --- | --- | --- | --- | --- | --- | --- |
| GO:0060337 | GO Biological Processes | type I interferon signaling pathway | 19 | 21.11 | -27.47 | -23.42 |
| GO:0001817 | GO Biological Processes | regulation of cytokine production | 21 | 23.33 | -12.38 | -9.07 |
| GO:0002757 | GO Biological Processes | immune response-activating signal transduction | 17 | 18.89 | -9.64 | -6.40 |
| GO:1903706 | GO Biological Processes | regulation of hemopoiesis | 15 | 16.67 | -9.47 | -6.26 |
| GO:1903901 | GO Biological Processes | negative regulation of viral life cycle | 8 | 8.89 | -8.91 | -5.79 |
| GO:0032623 | GO Biological Processes | interleukin-2 production | 7 | 7.78 | -8.39 | -5.38 |
| GO:0009617 | GO Biological Processes | response to bacterium | 13 | 14.44 | -5.52 | -2.88 |
| GO:0030260 | GO Biological Processes | entry into host cell | 6 | 6.67 | -4.96 | -2.41 |
| GO:0032480 | GO Biological Processes | negative regulation of type I interferon production | 4 | 4.44 | -4.55 | -2.08 |
| GO:0032655 | GO Biological Processes | regulation of interleukin-12 production | 4 | 4.44 | -4.24 | -1.84 |
| GO:0002683 | GO Biological Processes | negative regulation of immune system process | 9 | 10.00 | -4.10 | -1.73 |
| GO:0042832 | GO Biological Processes | defense response to protozoan | 3 | 3.33 | -3.91 | -1.60 |
| GO:0050690 | GO Biological Processes | regulation of defense response to virus by virus | 3 | 3.33 | -3.77 | -1.49 |
| GO:0002479 | GO Biological Processes | antigen processing and presentation of exogenous peptide antigen via MHC class I, TAP-dependent | 4 | 4.44 | -3.75 | -1.48 |
| GO:0051056 | GO Biological Processes | regulation of small GTPase mediated signal transduction | 7 | 7.78 | -3.74 | -1.47 |
| GO:0003724 | GO Molecular Functions | RNA helicase activity | 4 | 4.44 | -3.73 | -1.47 |
| GO:0008285 | GO Biological Processes | negative regulation of cell proliferation | 10 | 11.11 | -3.22 | -1.05 |
| GO:0070830 | GO Biological Processes | bicellular tight junction assembly | 3 | 3.33 | -2.90 | -0.78 |
| GO:0002366 | GO Biological Processes | leukocyte activation involved in immune response | 9 | 10.00 | -2.88 | -0.76 |
| GO:0031400 | GO Biological Processes | negative regulation of protein modification process | 8 | 8.89 | -2.61 | -0.53 |

Table S4. The KEGG pathway analysis of IRFs and neighbor genes in pancreatic carcinoma (Metascape).

| GO | Category | Description | Count | % | Log10(P) | Log10(q) |
| --- | --- | --- | --- | --- | --- | --- |
| hsa05162 | KEGG Pathway | Measles | 10 | 11.11 | -10.11 | -7.42 |
| hsa04660 | KEGG Pathway | T cell receptor signaling pathway | 4 | 4.44 | -3.22 | -1.47 |
| hsa05416 | KEGG Pathway | Viral myocarditis | 3 | 3.33 | -2.85 | -1.20 |
| hsa04514 | KEGG Pathway | Cell adhesion molecules (CAMs) | 4 | 4.44 | -2.68 | -1.16 |
| hsa05120 | KEGG Pathway | Epithelial cell signaling in Helicobacter pylori infection | 3 | 3.33 | -2.68 | -1.16 |
| hsa04612 | KEGG Pathway | Antigen processing and presentation | 3 | 3.33 | -2.52 | -1.03 |
| hsa04390 | KEGG Pathway | Hippo signaling pathway | 3 | 3.33 | -1.70 | -0.33 |
| hsa04530 | KEGG Pathway | Tight junction | 3 | 3.33 | -1.59 | -0.24 |

**
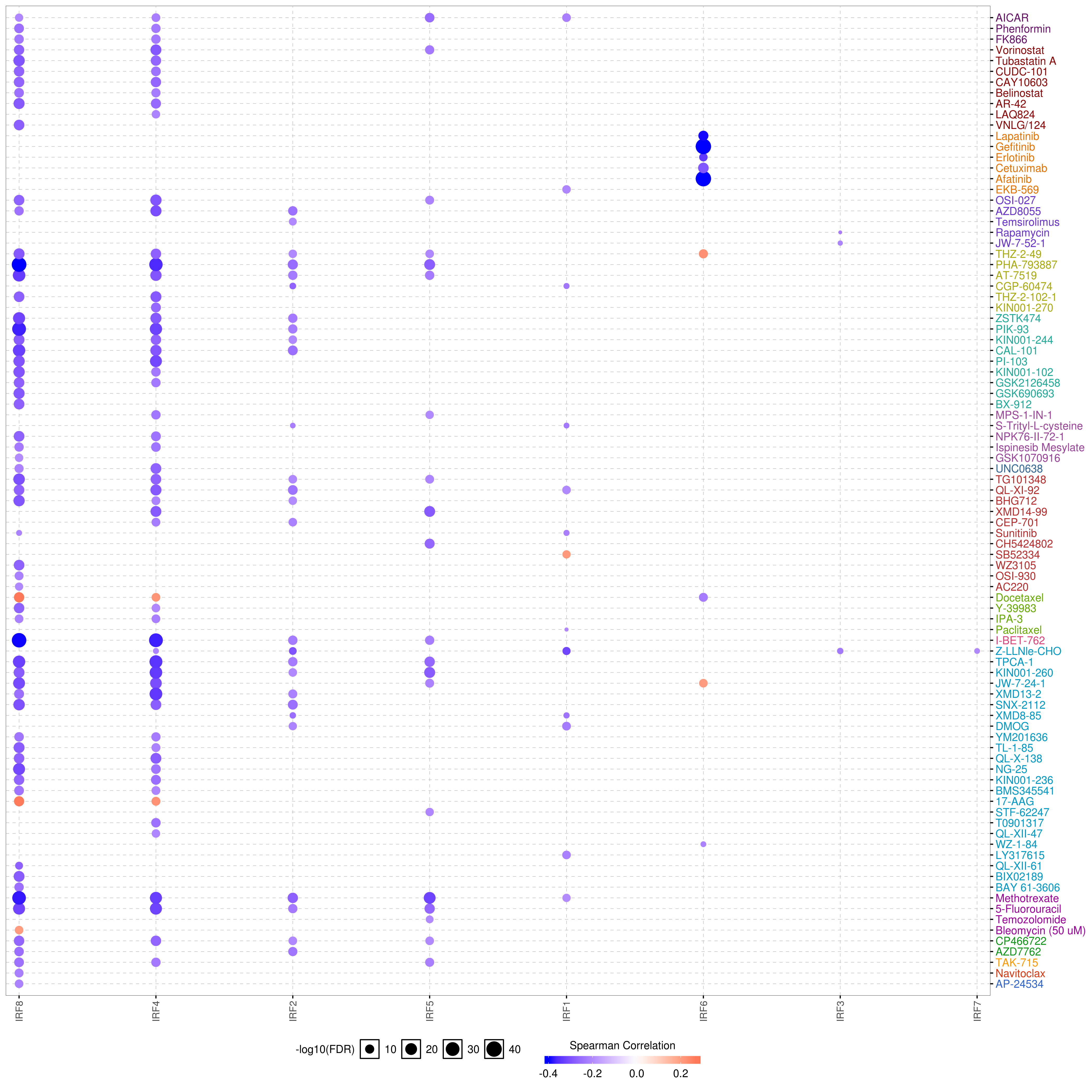
**

**Figure S1.** **The drug resistance analysis of IRFs according to GDSC IC50 drug data.** The Spearman correlation represent the gene expression correlates with the drug. The positive correlation means that the gene high expression is resistant to the drug, vise verse. This Figure was plotted using GSCALite (http://bioinfo.life.hust.edu.cn/web/GSCALite/).

**
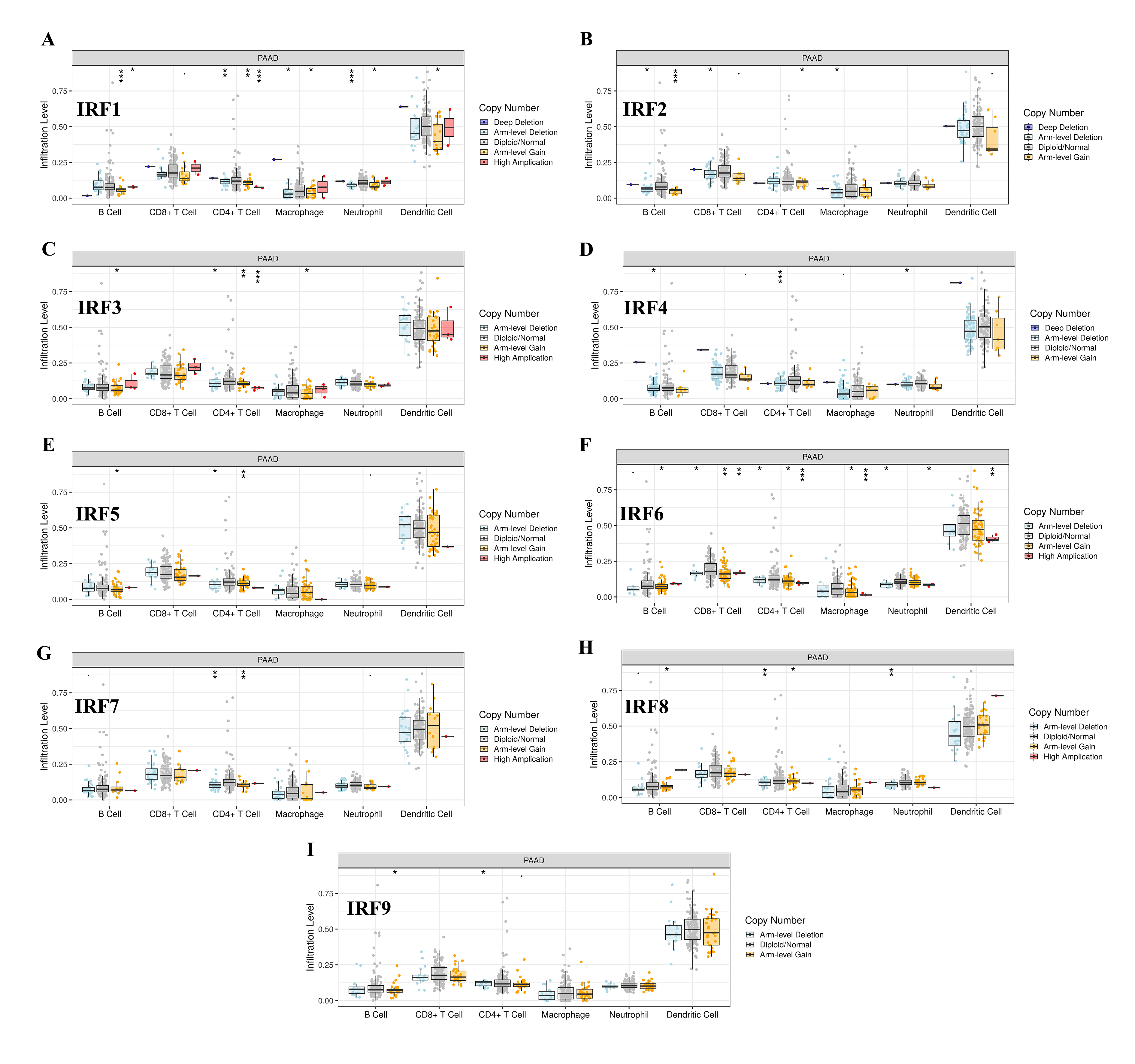
**

**Figure S2. the correlation between SCNA of IRFs and immune cell infiltration in pancreatic cancer.** The correlation between the SCNA of IRF1(A), IRF2(B), IRF3(C), IRF4(D), IRF5(E), IRF6(F), IRF7(G), IRF8(H), IRF9(I) and the abundance of B cells, CD8+ T cells, CD4+ T cells, Macrophage, Neutrophils and Dendritic cells. This Figure was plotted using TIMER (https://cistrome.shinyapps.io/timer/). SCNA, somatic copy number alterations; *P < 0.05, ** P < 0.01, *** P < 0.001.

**
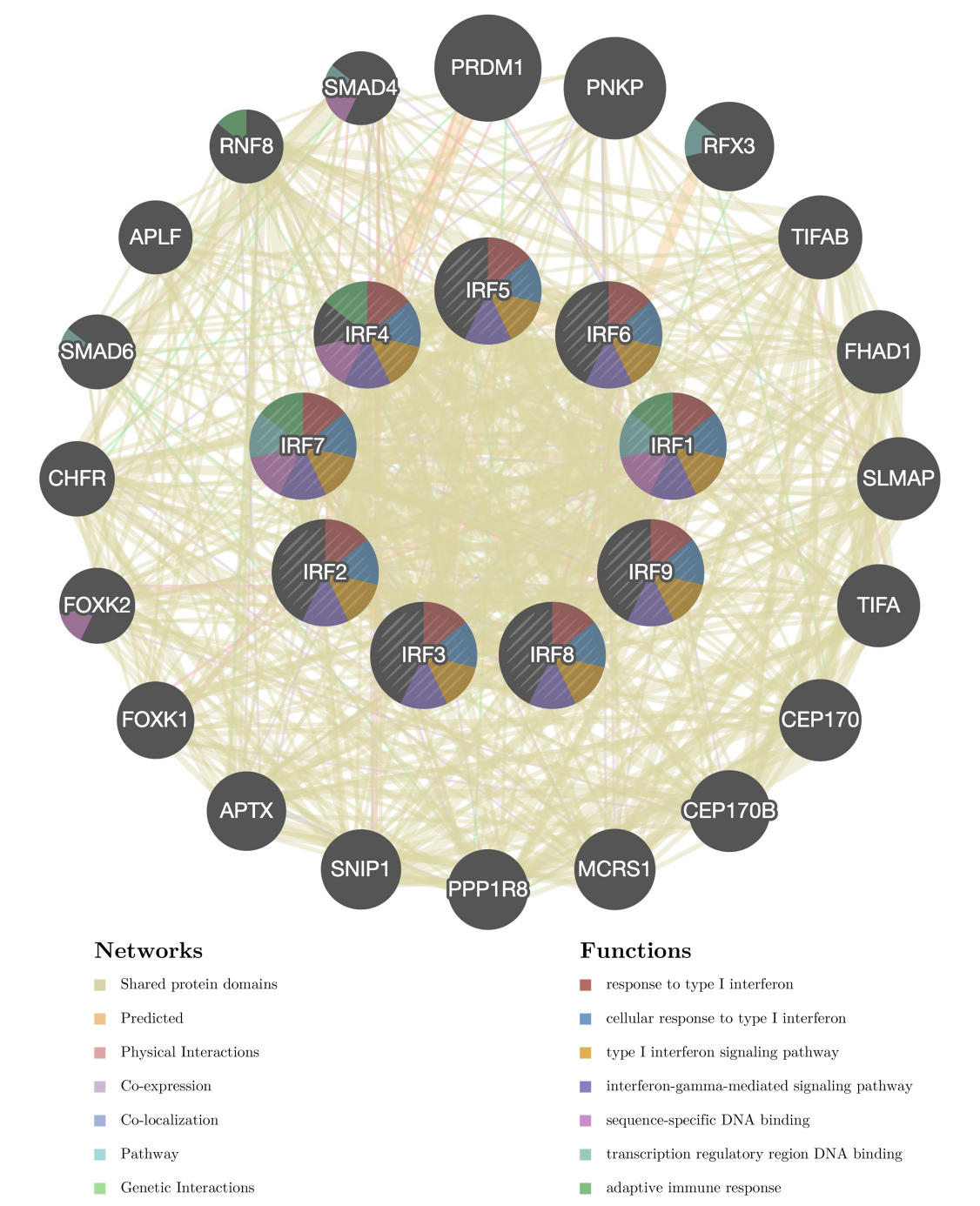
**

**Figure S3. Protein-protein interaction (PPI) network of IRFs.** PPI network and functional analysis indicating the gene set that was enriched in the target networks of IRFs. Different colors of the network edge indicate the bioinformatics methods applied: co-expression, website prediction, co-localization, shared protein domains, physical interaction, pathway and genetic interactions. The different colors for the network nodes indicate the biological functions of the set of enrichment genes. This Figure was plotted using GeneMANIA (http://genemania.org/).


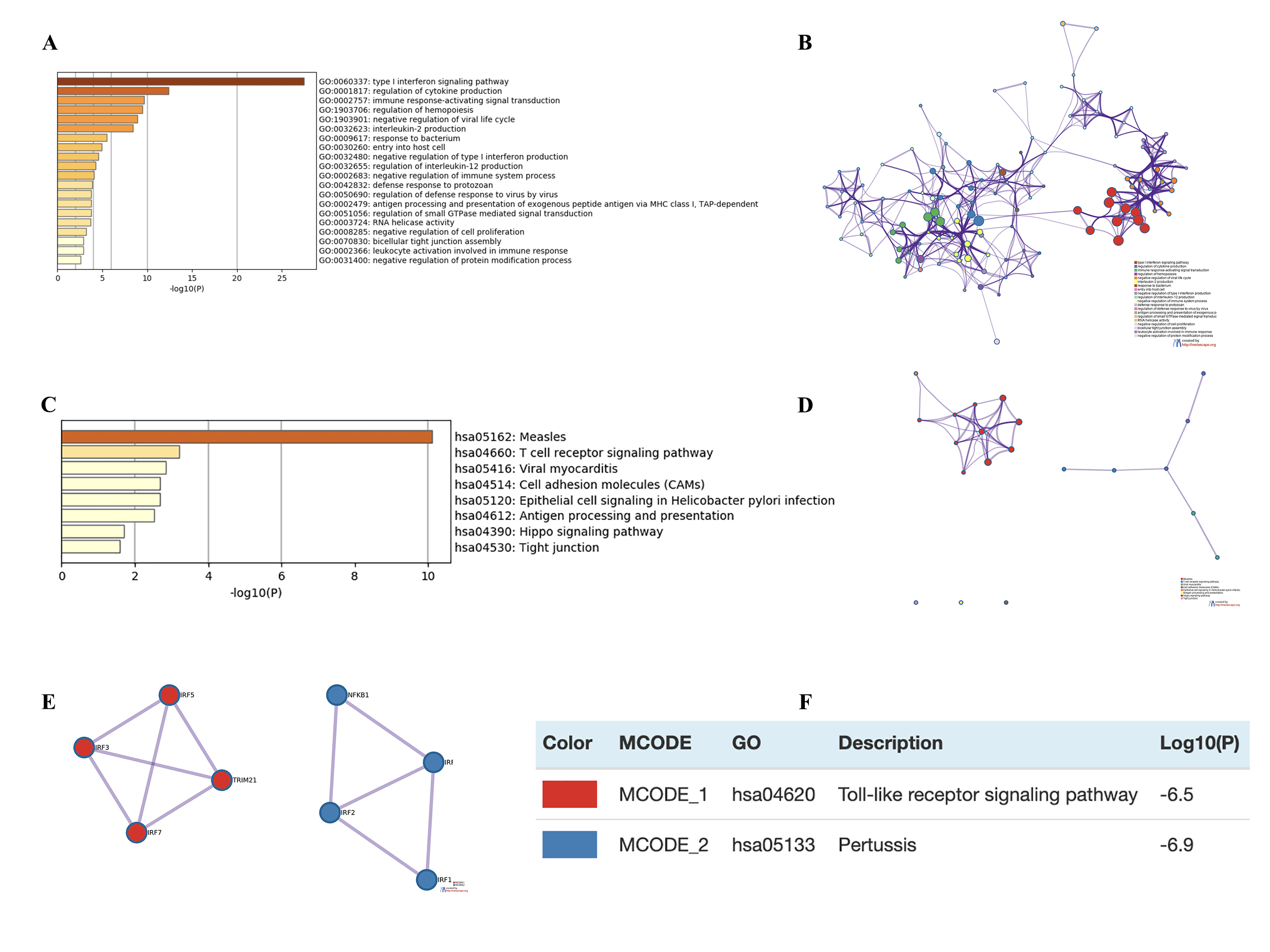


**Figure S4. The enrichment analysis of IRFs and neighboring gene**. (A) Heatmap of Gene Ontology (GO) enriched terms colored by enriched items. (B) Network of GO enriched terms colored by enriched items. (C) Heatmap of Kyoto Encyclopedia of Genes and Genomes (KEGG) enriched terms colored by enriched items. (D) Network of KEGG enriched terms colored by enriched items, (E) (PPI) network and four most significant MCODE components form the PPI network. (F) Independent functional enrichment analysis of three MCODE components. This Figure was plotted using Metascape (http://metascape.org).
